# Frameworks for nurturing and assessing students’ statistical thinking in regression modelling

**DOI:** 10.1186/2193-1801-3-S1-O5

**Published:** 2014-12-04

**Authors:** Ken W Li

**Affiliations:** Department of Information and Communications Technology, Hong Kong Institute of Vocational Education (Tsing Yi), Kragujevac, Hong Kong

## Background

One educational objective in any statistics curriculum is to teach students how to think statistically [1]. However, the definition of statistical thinking can be as broad as the thought processes involved in statistical activities or work or as narrow as focusing on the study of variation. To achieve this objective, it is necessary to know what statistical thinking means and how statistical thinking should be taught. In addition, it would be better to assess how well the students’ statistical thinking is developed. The aim of the assessment was to provide information to teachers about how to improve pedagogy to support learning [2].

## Methods

A test was conducted in a computing laboratory to assess key aspects of the students’ statistical thinking in regression modelling. A sample of 23 students studying in the Hong Kong Institute of Vocational Education attempted seven questions on an individual basis, and a set of real-life data with local context was given in the test. Questions 1-2, 3-5 and 6-7 were used to evaluate how much the students understand the given data regarding relations between variables, statistical relations between variables and functional relations between variables, respectively. According to Bishop and Talbot’s model of statistical thinking [3], the first two questions are equivalent to the task of reasoning about data, the fourth and the sixth questions are similar to the task of reasoning about results, and the last question is consistent with the task of reasoning about conclusions. A qualitative analysis of students’ responses to each of the questions was performed within the frameworks of Jones et al. [4] and Putt et al. [5] to determine which of these four levels of statistical thinking, idiosyncratic thinking, transitional thinking, quantitative thinking and analytical thinking, they achieved.

## Results

After the analysis was performed, it was found that a few students demonstrated the ability of idiosyncratic thinking by being able to justify the reasonableness and meaningfulness of data measurement with correct and thorough answers. Among the 23 students, about ten who demonstrated their good knowledge of correlation graphing and proficiency in using Excel graphing tools, displayed the ability of transitional thinking. Nine students showed the ability of quantitative thinking by accomplishing statistical hypothesis testing tasks in which they provided proper formulation of null and alternative hypotheses; correct statistical evidence and decision; and sound reasoning with statistical evidence from Excel output and statistical implications. None of students could deduce the data relation in a practical context, implying that the ability of analytical thinking was deficient.

## Conclusions

The findings of this study alert teachers to the importance of enhancing their students’ abilities of idiosyncratic and analytical thinking.
